# Effects of *Isaria cateniannulata* and *Beauveria bassiana* on Buckwheat Growth and Associated Insect Pest

**DOI:** 10.3390/biom15071039

**Published:** 2025-07-17

**Authors:** Xiaona Zhang, Lingdi Gu, Can Liu, Guimin Yang, Xue Yang, Kaifeng Huang, Qingfu Chen

**Affiliations:** 1 Research Center of Buckwheat Industry Technology, College of Life Science, Guizhou Normal University, Guiyang 550025, China; 201606003@gznu.edu.cn (X.Z.);; 2 School of International Education, Guizhou Normal University, Guiyang 550025, China

**Keywords:** *Isaria cateniannulata*, *Beauveria bassiana*, endophytic colonization, plant growth promotion, biocontrol, *Tetranychus urticae*

## Abstract

The *Tetranychus urticae* Koch (Acari: Tetranychidae) is one of the primary pests affecting buckwheat, and its management has become increasingly critical. Entomopathogenic fungi offer a promising way to solve this problem by providing both pest control and disease resistance, as well as promoting plant growth through endophytic colonization. This study investigated the effects of applying *Isaria cateniannulata* (Liang) Samson & Hywel-Jones and *Beauveria bassiana* (Bals.-Criv.) Vuill. on different buckwheat varieties, and analyzed the physiological indices of buckwheat, the population of *T. urticae* and *Euseius nicholsi* (Ehara & Lee). Results showed that the optimum concentration for fungal colonization on buckwheat was 1 × 10^7^ spores/mL. The combined application of *I. cateniannulata* and *B. bassiana* significantly enhanced buckwheat growth, with root length, plant height, main stem diameter, fresh weight, and dry weight reaching 63.3 mm, 24.1 cm, 2.1 mm, 2.0 g, and 0.1 g, respectively. The highest escape rate of *T. urticae* was 76.33%. Furthermore, the combined application of mixed fungal suspension and *E. nicholsi* had the best control effect on *T. urticae*, with pest suppression exceeding 97.83% and an oviposition as low as 0.25 eggs per female. This study is the first to demonstrate that the joint application of *I. cateniannulata* and *B. bassiana* can promote buckwheat growth and, when combined with predatory mites, effectively control *T. urticae.* These findings provide a theoretical basis for the development of integrated biocontrol strategies combining entomopathogenic fungi and predatory mites.

## 1. Introduction

Buckwheat is a member of the Polygonaceae family and the genus *Fagopyrum*. It is a versatile crop used both as food and medicine. Its nutritional profile, rich in micronutrients and high flavonoid content [1,2], makes it an essential food choice for populations with high dietary demands, weight management needs, and vegetarians [3,4]. With the increasing demand of consumers for buckwheat, the planting area of buckwheat has been expanding annually. However, due to its rapid growth and prolonged flowering period, buckwheat becomes an ideal food source for numerous pests, particularly in the seedling stage, with *Tetranychus urticae* Koch (Acari: Tetranychidae) being a primary pest. These mites feed on buckwheat leaves, disrupting the mesophyll cells and chloroplasts, resulting in chlorosis, yellowing, or even scorching of the leaves, which seriously impacts the yield of buckwheat [5,6]. Therefore, developing methods that can both control pests and promote buckwheat growth is of great significance for sustainable and safe buckwheat production.

Entomopathogenic fungi (EPF) are a unique group of fungi that can suppress pests and promote plant growth [7]. It has been reported that *Metarhizium anisopliae* (Metchn.) Sorokin, *Beauveria bassiana* (Bals.-Criv.) Vuill, *Isaria fumosorosea* Wize, *Isaria cateniannulata* (Liang) Samson & Hywel-Jones, *Isaria cicadae* (Miquel) Fr., and so on exhibit effective control against *T. urticae, with B. bassiana* and *I. cateniannulata* demonstrating the best control effects [8,9,10,11]. These two entomopathogenic fungi also have minimal adverse effects on the natural enemies of pest mites, such as *Neoseiulus pasteuri* (Athias-Henriot) and *Euseius nicholsi* (Ehara & Lee), which maintain relatively high survival rates after fungal treatment [12,13,14,15]

Beyond pest control, *B. bassiana* and *I. cateniannulata* can colonize plants endophytically, promoting growth across various crops. Their application has been linked to improvements in several plant morphological traits, including plant height, root and leaf development, biomass accumulation, and yield, in crops such as maize, tomato, and buckwheat [16,17,18,19]. However, whether two entomopathogenic fungi can be applied simultaneously, and whether their combined use yields additive or synergistic effects, remains unreported.

Therefore, this study intends to compare the effects of the combined application of *I. cateniannulata* and *B. bassiana* on buckwheat, *T. urticae*, and *E. nicholsi* through pot experiments. The goal is to identify optimal conditions that maximize buckwheat growth, effectively control *T. urticae,* and ensure safety for *E. nicholsi.* The findings will provide a theoretical basis and material foundation for the development of integrated biocontrol formulations.

## 2. Materials and Methods

### 2.1. Test Materials

Tested strains: *I. cateniannulata* No. 13 and *B. bassiana* No. 10, both isolated from Guizhou Lepidoptera pupae, are currently preserved in the Buckwheat Engineering Technology Research Center, College of Life Sciences, Guizhou Normal University.

Buckwheat: This study involved two species of buckwheat, *Fagopyrum esculentum* and *F. tataricum* (*Polygonaceae*). A total of six varieties were used: three of *F. tataricum* (No. M18, No. M13, No. M55) and three of *F. esculentum* (No. 1412-1, No. HT, No. 1412-16). These were preserved in the seed repository of the Buckwheat Engineering Technology Research Center, College of Life Sciences, Guizhou Normal University.

### 2.2. Formulation of Test Medium

Potato Dextrose Agar (PDA): PDA was prepared by boiling 200 g of peeled potato chips in distilled water for 30 min. The extract was filtered through four layers of gauze, and 20 g of glucose and 15 g of agar were added to the filtrate. After complete dissolution, the mixture was adjusted to a final volume of 1000 mL with distilled water and sterilized at 121 °C for 25 min. The sterilized medium was cooled to 50–60 °C, and 25 mL aliquots were poured into 90 mm diameter Petri dishes for use.

Potato Dextrose Broth (PDB): The preparation involved boiling 200 g of peeled potato chips in distilled water for 30 min, followed by filtration through four layers of gauze. Subsequently, 20 g of glucose was added, and the mixture was diluted to 1000 mL with distilled water. The broth was divided into 250 mL sterile Erlenmeyer flasks, each containing 150 mL of medium along with 8–10 glass beads to facilitate mixing. The flasks were sterilized at 121 °C for 25 min prior to use [20].

### 2.3. Preparation of Test Entomopathogenic Fungi Spore Suspension

The tested strains of *I. cateniannulata* and *B. bassiana* were respectively inoculated into Petri dishes containing PDA and incubated under controlled conditions at a temperature of 22 ± 1 °C, with a relative humidity of 75 ± 5%, and a photoperiod of L:D = 12:12 for 20 days, until the media were fully covered with fungal colonies. Selected colonies were then transferred into 150 mL of potato dextrose broth (PDB) in Erlenmeyer flasks and cultured on a rotary shaker at 150 rpm and 25 °C for 5 days. The spore suspension was prepared by filtering the fungal culture through double-layer sterile gauze. The resulting spore suspension was diluted with sterile water to concentrations of 1 × 10^8^, 1 × 10^7^, 1 × 10^6^, and 1 × 10^5^ spores/mL. Spore viability was assessed to be greater than 90% [14].

### 2.4. Planting of Tested Buckwheat

The test buckwheat was cultivated in pots with a diameter of 38 cm and a height of 26 cm, located within a greenhouse at the Buckwheat Engineering Technology Research Center of the College of Life Sciences, Guizhou Normal University. Each treatment consisted of 1000 pots, and 5 seeds sown per pot. The soil amount per pot was 7 kg, supplemented with 2 g of compound fertilizer (N:P:K = 15:15:15). Water and fertilizer conditions were standardized across all treatments. After sowing, the pots were watered every other day until the buckwheat plants reached 20 days of growth.

### 2.5. Feeding of Test Mites

*T. urticae* and *E. nicholsi* were reared in the insect breeding laboratory of the Buckwheat Engineering Technology Research Center, College of Life Sciences, Guizhou Normal University. A total of 50 *T. urticae* populations were reared on the tested buckwheat plants under controlled conditions of 22 ± 1 °C, 75 ± 5% relative humidity, and a L:D = 12:12 photoperiod. They were transferred to fresh, tested buckwheat plants every 10 days to ensure that each plant maintained a minimum of 50 adult female *T. urticae.*

*E. nicholsi* was reared on the same above buckwheat plants infested with *T. urticae,* with each plant supporting at least 5 *E. nicholsi* individuals. Both species were maintained under identical environmental conditions of 22 ± 1 °C, 75 ± 5% relative humidity, and an L:D = 12:12 photoperiod. Excess *E. nicholsi* individuals were transferred to new plants every 5 days.

### 2.6. Screening of the Concentration of Entomopathogenic Fungi with the Best Growth Promoting Effect on Buckwheat

A total of 120 uniformly grown buckwheat plants (M13) were selected from the sample described in Section 2.4. For each plant, three leaves, approximately 20 cm^2^ in area, were chosen. The spore suspensions of *I. cateniannulata* and *B. bassiana* at four concentrations (1 × 10^5^, 1 × 10^6^, 1 × 10^7^, and 1 × 10^8^ spores/mL) were sprayed on the abaxial surface of the leaves with a single-handed double-tube sprayer (0.2 mL per leaf) [21]. Five replicates were conducted for each treatment, with the control group sprayed with the same amount of distilled water. The plants were maintained under controlled conditions at a temperature of 22 ± 1 °C, a relative humidity of 75 ± 5%, and a photoperiod of L:D = 12:12, and were continuously cultured for 14 days. Physiological parameters, including root length, plant height, main stem diameter, leaf number, fresh weight, and dry weight (dried at 65 °C for 72 h), were measured to identify the optimal concentration of entomopathogenic fungal spore suspension for promoting buckwheat growth.

### 2.7. Screening for the Optimal Buckwheat Varieties and Entomogenous Fungal Strains for Promoting Buckwheat Growth

The spore suspensions of *I. cateniannulata* and *B. bassiana* at the optimal concentrations obtained in Section 2.6, as well as a mixed fungal formulation created by combining the two at a 1:1 volume ratio, were sprayed on buckwheat plants of different varieties (M13, M18, M55, 1412-1, 1412-16, and HT). Each plant was sprayed with the respective suspensions (0.2 mL per leaf). The control group was treated by spraying an equal volume of distilled water onto the leaves, with five replicates for each treatment. Following 14 days of continuous cultivation, multiple physiological parameters of the buckwheat plants were measured. These measurements were used to identify the buckwheat variety and entomopathogenic fungal strains that exhibit the most pronounced growth-promoting effects on buckwheat.

### 2.8. Screening for the Optimal Concentration of Entomopathogenic Fungi with the Highest Mortality Rate of T. urticae

Fresh buckwheat leaves with an area of about 20 cm^2^ were evenly coated with sterilized white Vaseline by spray method, and the petioles were wrapped with sterile absorbent cotton. The leaves were then placed in Petri dishes containing a layered setup comprising sponge, black cloth, and sterile transparent plastic film from bottom to top. Daily addition of sterile water to the absorbent cotton was performed to maintain leaf freshness. A total of 30 adult female *T. urticae* were gently transferred onto the abaxial surface of each leaf using a fine brush. Subsequently, 0.2 mL of each of the four spore suspension concentrations prepared in Section 2.3 was sprayed onto the leaves using a single-handed double-tube sprayer (0.2 mL per leaf), with five replicates per treatment. The control group was treated with sterile water, with all other conditions kept consistent. The cumulative death number of *T. urticae* was recorded for 5 consecutive days under the conditions of temperature 22 ± 1 °C, relative humidity 75 ± 5%, and photoperiod L:D = 12:12.

### 2.9. Determination of the Control Effect of Entomopathogenic Fungi Colonized Buckwheat Against T. urticae

A total of 15 buckwheat plants from each treatment group were selected 48 h after spraying with fungal suspensions or sterile water, as described in Section 2.7; 30 adult female mites were inoculated onto each leaf of each strain. Vaseline was applied around the leaf margins to prevent mite escape. The plants were then maintained under controlled conditions at 22 ± 1 °C, 75 ± 5% relative humidity, and a L:D = 12:12 photoperiod for 5 consecutive days. Daily observations were recorded, including the number of deaths, eggs laid, escaped mites, and fungal-infected mites.

### 2.10. Effects of Entomopathogenic Fungi-Colonized Buckwheat on the Behavior of Predatory Mites

A total of 15 strains from the experimental group (inoculated with *T. urticae* and sprayed with fungal suspensions) and 15 strains from the control group (inoculated with *T. urticae* and sprayed with sterile water) were selected. Five *E. nicholsi* were inoculated on the leaves of each strain (sterile water) and cultured continuously for 3 days. Daily observations were conducted to record the number of predatory mites, the number of *T. urticae*, and the infection status.

### 2.11. Statistical Analysis

Statistical analyses were performed using GraphPad Prism 9 and IBM SPSS Statistics 27.0. Data are presented as mean ± standard error (SE) from a minimum of three independent biological replicates. Normality was assessed using the Shapiro–Wilk test, and homogeneity of variances was verified with Levene’s test. For multiple comparisons among groups, one-way analysis of variance (ANOVA) was applied, followed by Tukey’s honestly significant difference (HSD) post hoc test. Statistical significance was defined as *p* < 0.05. In figures, different lowercase letters denote statistically significant differences (*p* < 0.05).

## 3. Results

### 3.1. The Concentration of Entomopathogenic Fungi with the Best Growth Promoting Effect on Buckwheat Was Obtained

**Figure 1:**
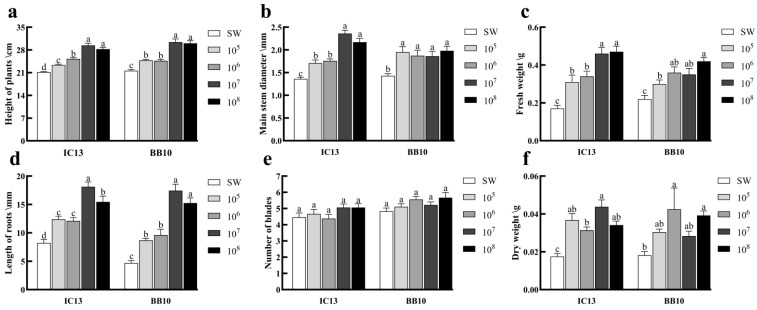
Effects of different concentrations of entomopathogenic fungi on growth indexes of buckwheat: (**a**) plant height; (**b**) main stem diameter; (**c**) fresh weight; (**d**) root length; (**e**) number of leaves; and (**f**) dry weight. Note: IC13, *I. cateniannulata* No. 13; BB10, *B. bassiana* No. 10; SW, sterile water. Error bars represent the standard error of the mean (SE). Different lowercase labels above each group indicate significant differences (one-way ANOVA, Tukey’s HSD post hoc test, *p* < 0.05) of group mean values.

After applying different concentrations of *I. cateniannulata* and *B. bassiana* spore suspension to the leaves of M13, all measured parameters—except for leaf number—were significantly higher in for leaf number which showed no significant difference compared to the control group (*p* > 0.05), were significantly higher than those of the control group (*p* < 0.05). The plant height (Figure 1a), main stem diameter (Figure 1b), and fresh weight (Figure 1c) exhibited a gradual increase with rising spore concentrations. Root length initially increased and then decreased with increasing spore concentration (Figure 1d), reaching a maximum at 1 × 10^7^ spores/mL, 2.2 times greater than the control group.

At a concentration of 1 × 10^7^ spores/mL, both *I. cateniannulata* and *B. bassiana* exhibited optimal growth-promoting effects on M13. *I. cateniannulata* increased plant height (Figure 1a), main stem diameter (Figure 1b), fresh weight (Figure 1c), root length (Figure 1d), and dry weight (Figure 1f) by 1.39 times, 1.74 times, 2.71 times, 2.20 times, and 2.50 times, respectively, compared with the control group. *B. bassiana* enhanced plant height (Figure 1a), main stem diameter (Figure 1b), and root length (Figure 1d) by 1.41 times, 1.30 times, and 3.71 times, respectively, compared with the control group.

### 3.2. Effects of Different Entomopathogenic Fungi Strains on the Growth of Different Buckwheat Varieties

**Figure 2:**
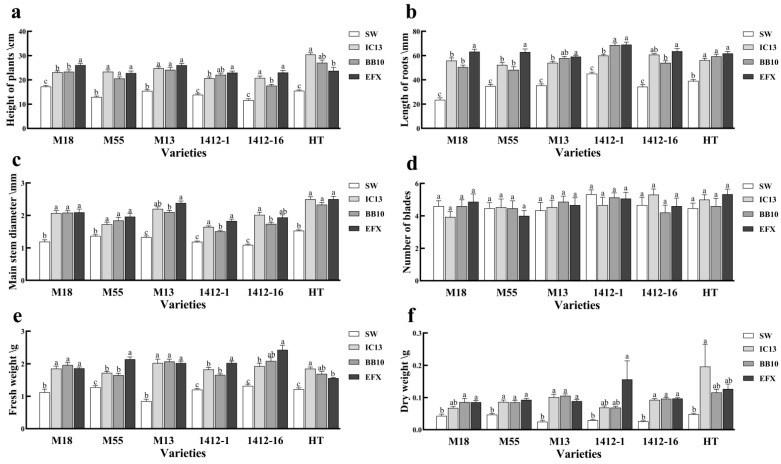
Effects of *I. cateniannulata*, *B. bassiana*, and mixed fungal suspension on the growth of different buckwheat seedling lines: (**a**) plant height; (**b**) root length; (**c**) main stem diameter; (**d**) number of leaves; (**e**) fresh weight; and (**f**) dry weight. Note: IC13, *I. cateniannulata* No. 13; BB10, *B. bassiana* No. 10; EFX, mixed fungal suspension; SW, sterile water; M18, *F. tataricum* No. M18; M13, *F. tataricum* No. M13; M55, *F. tataricum* No. M55; 1412-1, *F. esculentum* No. 1412-1; 1412-16, *F. esculentum* No. 1412-16; HT, *F. esculentum* No. HT. Error bars represent the standard error of the mean (SE). Different lowercase labels above each group indicate significant differences (one-way ANOVA, Tukey’s HSD post hoc test, *p* < 0.05) of group mean values.

After spraying the optimum concentration of *I. cateniannulata*, *B. bassiana*, and mixed fungal suspension on buckwheat, all growth parameters—except for leaf number, which showed no significant difference compared to the control—were significantly higher in the treatment groups. Particularly, plant height (Figure 2a), root length (Figure 2b), main stem diameter (Figure 2c), fresh weight (Figure 2e), and dry weight (Figure 2f) of buckwheat in the experimental group were higher than those in the control group.

**Figure 3:**
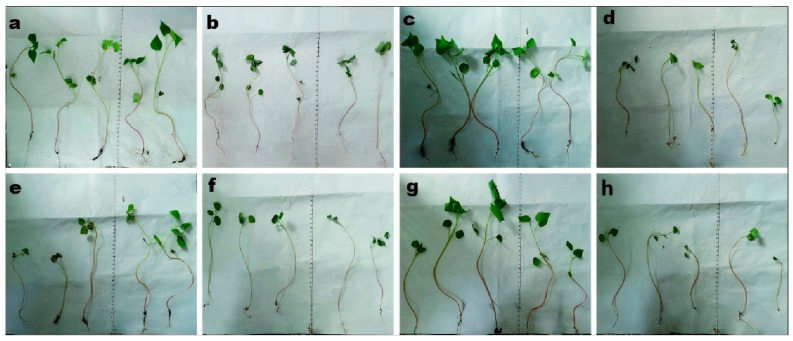
Effects of different strains on the growth of buckwheat seedlings: (**a**) *F. esculentum* + *B. bassiana*; (**b**) *F. esculentum* + *I. cateniannulata*; (**c**) *F. esculentum* + mixed fungal suspension; (**d**) *F. esculentum* + sterile water (Control); (**e**) *F. tataricum* + *B. bassiana*; (**f**) *F. tataricum* + *I. cateniannulata*; (**g**) *F. tataricum* + mixed fungal suspension; (**h**) *F. tataricum* + sterile water (Control).

Different fungal strains exhibited varying effects on the growth of different buckwheat cultivars. The combined fungal suspension produced the most pronounced plant height (Figure 2a) promotion in M18 and 1412-1, with increases of 1.51 and 1.66 times compared to the control group. It also showed the greatest enhancement in root length (Figure 2b) across all varieties, with increases of 2.69, 1.80, 1.67, 1.53, and 1.85 times compared to the control. The M13 and 1412-1 showed the greatest enhancement in main stem diameter (Figure 2c), with rises of 1.79 and 1.54 times compared to the control. Additionally, 1412-1 and 1412-16 exhibited the strongest effects on fresh weight (Figure 2e), which were 1.69, 1.84, and 5.30 times compared to the control group. These differences are likely due to the distinct metabolic characteristics and physiological functions, which can affect the growth and development of buckwheat. The plant height, fresh weight, and dry weight of HT exhibited the most significant increases, with values 1.92, 1.50, and 3.80 times higher than those of the control group, respectively. Additionally, the main stem diameter of cultivar 1412-16 was the greatest, being 1.88 times that of the control. These differences were also visually evident in seedling morphology and vigor, where the seedlings treated with mixed fungal suspension appeared more robust and vigorous (Figure 3c,g).

### 3.3. The Concentration of Entomopathogenic Fungi with the Highest Mortality to T. urticae Was Obtained

**Figure 4:**
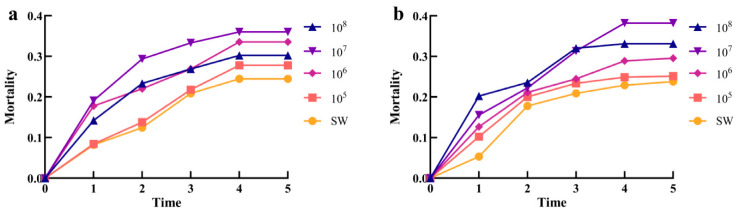
The lethal rate of different concentrations of entomopathogenic fungi on *T. urticae*: (**a**) *I. cateniannulata* treatment; (**b**) *B. bassiana* treatment.

After *I. cateniannulata* and *B. bassiana* infected *T. urticae*, the mortality of mites increased gradually with time, and reached the maximum on the 4th day, with the mortality of 36 ± 1% (Figure 4a) and 38 ± 1% (Figure 4b), respectively. At this time, the optimal concentration of fungal suspension solution was 1 × 10^7^ spores/mL, which was due to the synergistic effect of fungal colonization intensity and plant immune response in a certain concentration range, and gradually increased with increasing concentration.

### 3.4. Effects of Entomopathogenic Fungi Colonization on the Population Growth of T. urticae

**Figure 5:**
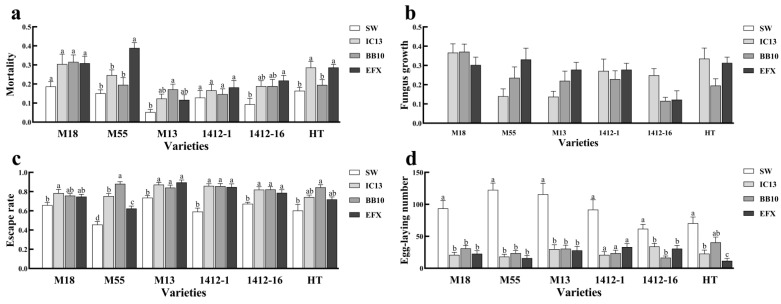
Effects of colonization of *I. cateniannulata* and *B. bassiana* on population dynamics of *T. urticae*: (**a**) mortality rate of *T. urticae*; (**b**) fungus growth rate of *T. urticae*; (**c**) escape rate of *T. urticae*; (**d**) egg-laying number of *T. urticae*. Note: IC13, *I. cateniannulata* No. 13; BB10, *B. bassiana* No. 10; EFX, mixed fungal suspension; SW, sterile water; M18, *F. tataricum* No. M18; M13, *F. tataricum* No. M13; M55, *F. tataricum* No. M55; 1412-1, *F. esculentum* No. 1412-1; 1412-16, *F. esculentum* No. 1412-16; HT, *F. esculentum* No. HT. Error bars represent the standard error of the mean (SE). Different lowercase labels above each group indicate significant differences (one-way ANOVA, Tukey’s HSD post hoc test, *p* < 0.05) of group mean values.

The mortality (Figure 5a), fungus growth rate (Figure 5b), and escape rate (Figure 5c) of *T. urticae* were higher than those of the control group, and the number of eggs laid was lower than that of the control group (Figure 5d).

Different strains colonized different buckwheat varieties had different effects on *T. urticae*. The mortality of M55 and 1412-16 to *T. urticae* was the highest, which was 2.60 and 2.33 times higher than that of the control group. The mortality rate of M13 colonized by *B. bassiana* to *T. urticae* was the highest, which was 3.27 times higher than that of the control group (Figure 5a). The escape rate of M18 colonized by *I. cateniannulata* to *T. urticae* was the highest, which was 1.18 times higher than that of the control group (Figure 5c). The escape rates of M55 and HT colonized by *B. bassiana* to *T. urticae* were the highest, which were 1.92 and 1.40 times that of the control group, respectively. The colonization of HT by the mixed fungal suspension had the greatest effect on the number of eggs laid by *T. urticae*, which was only 0.16 times that of the control group.

### 3.5. Effects of Entomopathogenic Fungi Colonization on the Behavior of E. nicholsi

**Figure 6:**
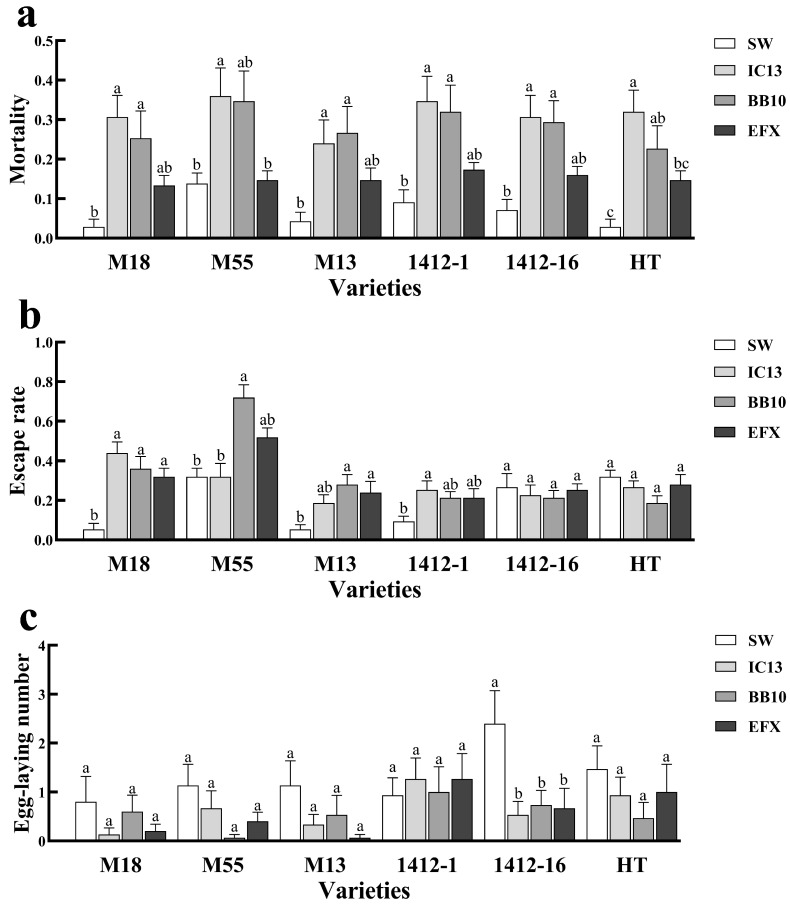
Effects of colonization of *I. cateniannulata* and *B. bassiana* on the population dynamics of *E. nicholsi*: (**a**) mortality rate of *E. nicholsi*; (**b**) escape rate of *E. nicholsi;* (**c**) egg-laying number of *E. nicholsi*. Note: IC13, *I. cateniannulata* No. 13; BB10, *B. bassiana* No. 10; EFX, mixed fungal suspension; SW, sterile water; M18, *F. tataricum* No. M18; M13, *F. tataricum* No. M13; M55, *F. tataricum* No. M55; 1412-1, *F. esculentum* No. 1412-1; 1412-16, *F. esculentum* No. 1412-16; HT, *F. esculentum* No. HT. Error bars represent the standard error of the mean (SE). Different lowercase labels above each group indicate significant differences (one-way ANOVA, Tukey’s HSD post hoc test, *p* < 0.05) of group mean values.

After planting of buckwheat treated with *I. cateniannulata*, *B. bassiana*, and mixed fungal suspension, the escape rate and mortality of *E. nicholsi* in the experimental group were higher than those in the control group, while the number of eggs laid was reduced. The mixed fungal suspension exhibited the least effect on *E. nicholsi* mortality (Figure 6), which could be due to the relatively low virulence of the combined strains, or this mutual inhibition would also have occurred in pathogenicity against *T. urticae*.

After colonization of buckwheat with different fungal strains, the escape rate and mortality of *E. nicholsi* showed varying responses (Figure 6a,b). Among all treatments, colonization with the mixed fungal suspension in cultivar M55 exhibited the weakest effect on mortality of *E. nicholsi*, resulting in only a 1.06 times increase compared to the control group. Similarly, colonization with *I. cateniannulata* in M55 and the mixed fungal suspension in 1412-1 had a limited impact on the escape rate of *E. nicholsi*, which were 1.03 and 2.33 times higher than that of the control group, respectively. Notably, the number of eggs laid by *E. nicholsi* was highest when 1412-1 was colonized with *B. bassiana* (Figure 6c).

### 3.6. Effects of Entomopathogenic Fungi and E. nicholsi on Population Behavior of T. urticae

**Figure 7:**
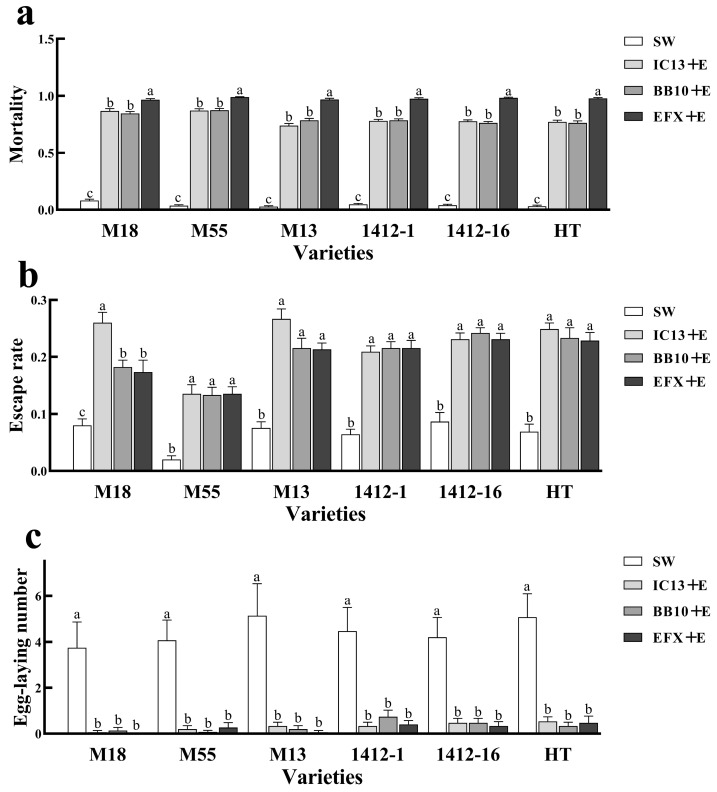
Dynamic changes in the effects of *I. cateniannulata*, *B. bassiana*, and *E. nicholsi* on the growth of *T. urticae*: (**a**) mortality rate of *T. urticae*; (**b**) escape rate of *T. urticae*; (**c**) egg-laying number of *T. urticae*. Note: IC13 + E, *I. cateniannulata* and *E. nicholsi*; BB10 + E, *B. bassiana* and *E. nicholsi*; EFX + E, mixed fungal suspension and *E. nicholsi*; SW, sterile water. Error bars represent the standard error of the mean (SE). Different lowercase labels above each group indicate significant differences (one-way ANOVA, Tukey’s HSD post hoc test, *p* < 0.05) of group mean values.

Under the combined influence of *I. cateniannulata*, *B. bassiana*, and *E. nicholsi*, the mortality (Figure 7a) and escape rate (Figure 7b) of *T. urticae* in the experimental group were higher than those in the control group, and the number of eggs laid (Figure 7c) was lower than that in the control group. The mortality rate of the mixture of mixed fungal suspension and *E. nicholsi* to *T. urticae* was the highest, which was 2.29 times compared to the control group, followed by single colony and *E. nicholsi*, which was 1.99 times that of the control group. The escape rate of *E. nicholsi* from *T. urticae* was highest when combined with the mixed fungal suspension, reaching 2.83 times that of the control group, while the number of eggs laid was significantly lower, at only 0.08 times that of the control.

## 4. Discussion

The colonization of entomopathogenic fungi and their promotion of plant growth have become a current research hotspot. Both *Beauveria brongniartii* and *Metarhizium brunneum* have been shown to increase various growth parameters in common bean, including plant height, leaf number, root fresh weight, and aboveground biomass [22]. *Beauveria brongniartii* enhanced tobacco seedling leaf count, plant height, root length, photosynthetic rate, chlorophyll content, and stomatal size [23]. *I. cateniannulata* increased buckwheat sprout length, sprout diameter, germination rate, and yield [24]. In this study, the co-application of *I. cateniannulata* and *B. bassiana* to buckwheat synergistically improved root length, plant height, stem diameter, fresh weight, and dry weight. This is likely due to the secretion of plant growth regulators such as indole acetic acid (IAA) and gibberellin (GA) by the fungi, which can directly stimulate cell division and elongation [25].

Concurrently, the dual role of entomopathogenic fungi in promoting plant growth while simultaneously enhancing plant resistance to pests has also become a research focus [26,27]. The core mechanism is that entomopathogenic fungi can enhance plant resistance by activating the jasmonic acid (JA) and salicylic acid (SA) defense signaling pathways, thereby inducing systemic resistance (ISR). In addition, they can elevate the activities of antioxidant enzymes such as peroxidase (POD) and superoxide dismutase (SOD), which collectively strengthen the plant’s chemical defense against herbivorous pests [28,29]. Endophytic colonization of sorghum by *Metarhizium robertsii* reduced larval infestation levels of the stalk borer *Sesamia nonagrioides* [30]. *Isaria fumosorosea* colonizing sweet pepper increased *Myzus persicae* mortality and shortened average survival time [31]. Similarly, *Beauveria brongniartii* colonization of horse chestnut trees reduced larval length and width of *Cameraria ohridella* [32], while *Purpureocillium lilacinum* in cotton decreased *Helicoverpa zea* larval survival and prolonged pupation time [33]. This study corroborates these findings, demonstrating that *I. cateniannulata* and *B. bassiana* colonization significantly increased *T. urticae* mortality, which confirms that their colonization could improve the control effect of buckwheat on *T. urticae*. The improved pest control efficacy is primarily attributed to the enhancement in plant systemic resistance induced by fungal colonization [34], which subsequently increases the mortality of Tetranychus urticae and triggers behavioral changes such as increased escape activity and reduced oviposition [35,36].

At present, few studies have investigated the combined use of entomopathogenic fungi colonizing plants and predatory mites for pest control. After *B. bassiana* colonized eggplant plants, it was applied together with the predatory mite *Stratiolaelaps scimitus*, exhibiting a synergistic effect in controlling *Frankliniella occidentalis* and significantly reducing both larval and adult populations [37]. Combined application of *Metarhizium anisopliae* and predatory mites *Neoseiulus californicus* for biological control of *T. urticae* on chrysanthemum reduced *T. urticae* populations compared with the control treatment [38]. The combined application of the entomopathogenic fungus *Metarhizium anisopliae* and the predatory soil mite *Gaeolaelaps aculeifer* significantly increased the mortality of *Lycoriella auripila* larvae [39]. In this study, the co-application of entomopathogenic fungi and predatory mites following fungal colonization of plants showed enhanced efficacy, with the combination of the mixed fungal suspension and predatory mites achieving the best control effect. This synergistic enhancement may involve multiple mechanisms, such as fungal colonization improving the predatory environment for natural enemies or indirectly influencing pest behavior, as well as plant volatiles potentially attracting natural enemies [36,40,41,42,43,44].

## 5. Conclusions

This study was the first to colonize buckwheat with the combined application of *I. cateniannulata* and *B. bassiana* at a concentration of 1 × 10^7^ spores/mL. The treatment demonstrated the most significant promotion of physiological indices of buckwheat, including root length, plant height, main stem diameter, fresh weight, and dry weight. Furthermore, the combined application of the mixed fungal suspension and *E. nicholsi* exhibited the most effective control of *T. urticae* and showed the highest safety profile for predatory mites.

## Data Availability

The data that support the findings of this study are available from the corresponding author and the first author upon reasonable request.
